# Environmental Factors Associated with Disease Progression after the First Demyelinating Event: Results from the Multi-Center SET Study

**DOI:** 10.1371/journal.pone.0053996

**Published:** 2013-01-08

**Authors:** Dana Horakova, Robert Zivadinov, Bianca Weinstock-Guttman, Eva Havrdova, Jun Qu, Miriam Tamaño-Blanco, Darlene Badgett, Michaela Tyblova, Niels Bergsland, Sara Hussein, Laura Willis, Jan Krasensky, Manuela Vaneckova, Zdenek Seidl, Petra Lelkova, Michael G. Dwyer, Ming Zhang, Haoying Yu, Xiaotao Duan, Tomas Kalincik, Murali Ramanathan

**Affiliations:** 1 Department of Neurology and Center of Clinical Neuroscience, Charles University in Prague, 1st Faculty of Medicine and General University Hospital, Charles University, Prague, Czech Republic; 2 Department of Neurology, State University of New York, Buffalo, New York, United States of America; 3 Buffalo Neuroimaging Analysis Center, Department of Neurology, State University of New York, Buffalo, New York, United States of America; 4 Department of Pharmaceutical Sciences, State University of New York, Buffalo, New York, United States of America; 5 Department of Radiology, 1st Faculty of Medicine and General University Hospital, Charles University, Prague, Czech Republic; 6 Department of Pediatrics, 1st Faculty of Medicine and General University Hospital, Charles University, Prague, Czech Republic; 7 Melbourne Brain Centre, Faculty of Medicine, Dentistry and Health Sciences, University of Melbourne, Victoria, Australia; University of Utah School of Medicine, United States of America

## Abstract

**Objectives:**

To investigate the associations of environmental MS risk factors with clinical and MRI measures of progression in high-risk clinically isolated syndromes (CIS) after the first demyelinating event.

**Methods:**

We analyzed 211 CIS patients (age: 28.9±7.8 years) enrolled in the SET study, a multi-center study of high-risk CIS patients. Pre-treatment samples were analyzed for IgG antibodies against cytomegalovirus (anti-CMV), Epstein Barr virus (EBV) early nuclear antigen-1 (EBNA-1), viral capsid antigen (VCA), early antigen-diffuse (EA-D), 25 hydroxy-vitamin D3 and cotinine levels and HLA DRB1*1501 status. The inclusion criteria required evaluation within 4 months of the initial demyelinating event, 2 or more brain MRI lesions and the presence of two or more oligoclonal bands in cerebrospinal fluid. All patients were treated with interferon-beta. Clinical and MRI assessments were obtained at baseline, 6, 12, and 24 months.

**Results:**

The time to first relapse decreased and the number of relapses increased with anti-CMV IgG positivity. Smoking was associated with increased number and volume of contrast-enhancing lesions (CEL) during the 2-year period. The cumulative number of CEL and T2 lesions during the 2-year period was greater for individuals in the highest quartile of anti-EBV VCA IgG antibodies. The percent loss of brain volume was increased for those in the highest quartile of with anti-EBV VCA IgG antibodies.

**Conclusions:**

Relapses in CIS patients were associated with CMV positivity whereas anti-EBV VCA positivity was associated with progression on MRI measures, including accumulation of CEL and T2 lesions and development of brain atrophy.

## Introduction

Multiple sclerosis (MS) is a chronic inflammatory demyelinating disease of the brain and spinal cord that causes physical and cognitive disability. Disease progression in MS is mediated by inflammatory and neurodegenerative processes that cause injury to the brain and spinal cord.

Epstein-Barr virus (EBV) exposure, decreased vitamin D levels and smoking are the best-established environmental risk factors for MS [1]–[3]. Efforts to integrate environmental risk factors with the risk contributions of genetic variations such HLA DRB1*1501 have been reported [3], [4].

Cross-sectional studies suggest that exposure to EBV, cigarette smoking and low vitamin D are also associated with MS disease progression. Higher levels of EBV antibodies are associated with greater brain atrophy [4]–[6]. Smoking increases lesion volume and the risk of developing secondary-progressive MS [7]–[9] whereas increased vitamin D levels have been associated with fewer relapses [10]–[13].

The first clinical demyelinating event, often known as clinically isolated syndrome (CIS), represents a crucial opportunity for understanding the factors involved in conversion to MS. The environmental factors in CIS have not been extensively investigated prospectively and their role in MS disease course and progression is not well characterized. Anti-EBV nuclear antigen (EBNA-1) antibodies were associated with progression to clinically definite MS (CDMS) and with the formation of new lesions [14] and smoking has been linked to increased risk of developing CDMS and to decreased time to first relapse [15]. Here, we examine the relationship between multiple environmental risk factors for MS and disease progression as assessed by clinical and MRI measures obtained *longitudinally* in a cohort of CIS patients after the initial demyelinating event in a controlled, multi-center, observational study. Drawing on the body of prior research, pre-treatment serum from this CIS cohort was assessed for anti-EBV EBNA-1, anti-EBV viral capsid antigen (VCA), anti-cytomegalovirus (CMV) antibodies, vitamin D levels and active smoking status via cotinine levels. All patients were initiated on intramuscular interferon beta-1a (AVONEX®) therapy at time of entry.

## Methods

### Study Population

#### Study Setting

Multi-center, prospective, longitudinal observational study.

#### Clinical Study Design

The Observational Study of Early Interferon beta 1-a Treatment in High Risk Subjects after CIS (SET study) is a prospective observational clinical study coordinated by the Charles University in Prague. It involves eight centers from the Czech Republic.

The objective of the SET study is to determine clinical and MRI predictors of response to interferon beta 1-a therapy in CIS. All patients are treated with 30 µg, intramuscular interferon beta 1-a (AVONEX®). The trial includes clinical visits every 3-months for 4 years and subsequent long term follow up in routine clinical practice. A range of clinical and MRI outcomes (including time to CDMS, disability progression, quality of life measures and yearly volumetric MRI scans) were obtained longitudinally.

#### Study Population

The study population were CIS patients with the following characteristics: 18–55 years of age, enrolled within 4 months from the clinical event, EDSS ≤3.5, presence of ≥2 T2-hyperintense lesions on diagnostic MRI, and presence of ≥2 oligoclonal bands in CSF obtained at the screening visit prior to steroid treatment. All patients were treated with 3–5 g of methylprednisolone for the first symptom and baseline MRI was performed at least 30 days after steroid administration.

Of the 220 CIS patients enrolled in the SET study, 216 CIS patients had available clinical follow-up and MRI data. This analysis was limited to 211 subjects with environmental factor biomarkers or HLA DRB1*1501 genotyping available (Results S1).

Clinical and MRI assessments were obtained at baseline, 6, 12 and 24 months. Clinical assessments were performed using the Kurtzke Expanded Disability Status Scale (EDSS). In case of relapses, patients were evaluated within 4 days from onset of the new symptoms.

#### Ethics Statement

The Medical Ethics Committees of the General University Hospital and 1st Faculty of Medicine of Charles University, Prague, Czech Republic, approved the study protocol and the informed consent procedure. In addition, approvals were obtained from local medical ethics committees of all other participating centers (KZ Hospital, Teplice; University Hospitals in Brno, Plzen and Olomouc; St. Anne's University Hospital, Brno; Motol University Hospital, Prague and Kralovske Vinohrady University Hospital, Prague). Written informed consent was obtained from all patients at enrolment.

### Environmental Factors

The technicians conducting analyses of anti-EBV and anti-CMV antibodies, smoking status, vitamin D and HLA DRB1*1501 status were blinded to the patients' clinical status. All analyses were conducted in serum samples obtained at the screening visit prior to any corticosteroid or interferon beta-1a treatment.

#### Anti-EBV and Anti-CMV antibodies

Enzyme-linked immunosorbent assay (ELISA) kits from Diamedix Corporation (Miami, FL) were used to quantify anti-CMV IgG, anti-EBV VCA, EBNA-1 and early antigen (EA-D) IgG antibodies. Serial dilutions of positive control samples provided with each kit were used to generate standard curves. The anti-CMV, EBNA-1 and VCA IgG levels were normalized to the manufacturer's cut-off calibrator standard, which represents a sample that is just positive. Anti-CMV and anti-EBV antibody levels were available for 193 patients. The anti-EBNA-1 and anti-VCA relative concentrations were categorized into quartiles.

#### Smoking Status

Cotinine levels were measured using a validated liquid chromatography-mass spectrometry method. A cotinine level threshold of 10 ng/ml was used to categorize subject as active smokers [16], [17]. Cotinine levels were available for 194 patients.

#### Vitamin D Levels

The vitamin D metabolite 25 hydroxy vitamin D3 (25(OH)VD_3_) was measured using liquid chromatography-tandem mass spectrometry (LC-MS/MS) methods with stable-isotope-labeled internal standards as previously described [18].

Raw 25(OH)VD_3_ levels were deseasonalized using sinusoidal regression [19] and dichotomized based on the clinical threshold for vitamin D deficiency (25(OH)VD_3_ level <20 ng/ml) [20]. Levels of 25(OH)VD_3_ were available for 185 patients. The missing subjects lacked serum or had insufficient serum.

### Genotyping

HLA DRB1*1501 status was obtained by genotyping DNA from peripheral blood for *rs3135005*, a SNP strongly correlated with HLA DRB1*1501 status, using allele discrimination (Applied Biosystems, Redwood City, CA) [21]. HLA DRB1*1501 status was available for 198 patients. The missing genotypes were due to non-availability of DNA samples or ambiguous genotypes.

### MRI Acquisition and Analysis

#### Image Acquisition

MRI was performed on all patients using a 1.5 T magnet (Philips Gyroscan NT 15, Best, the Netherlands). Acquisition details are in Results S1.

#### Image Analysis

The scans were collected centrally at the Department of Radiology at Charles University (Prague, Czech Republic). All MRI scans were transferred analyzed by the Buffalo Neuroimaging Analysis Center (Buffalo, New York, USA).

#### Lesion Measures

The T2- and contrast enhancing lesion (CEL) number and lesion volumes (LVs) were measured as previously described [22]. For each time point, the identification of new and enlarging T2-lesions was performed via a “subtraction image” methodology (See Results S1).

#### Global and Tissue-Specific Atrophy Measures

For baseline analyses, SIENAX software was used (version 2.6). Normalized whole brain volume (WBV), normalized gray matter volume (NGMV) and normalized white matter volume (NWMV) were measured as previously described [23]. For longitudinal changes of the WBV, we used the SIENA method [24] to calculate the percentage brain volume change (PBVC). To quantify longitudinal GM and WM volume changes, we used a modified hybrid of FMRIB's SIENA and SIENAX tools (See Results S1).

### Data Analysis

SPSS (IBM Inc., Armonk, NY, version 19.0) statistical program was used for all statistical analyses. In view of the multiple testing, a conservative *p*-value of ≤0.01 was used to assess significance; *p*-values ≤0.05 were considered to be trends. The clinical variables were analyzed with appropriate regression analyses that included age, sex and the genetic or environmental variable of interest as predictors. The regression models for MRI progression variables additionally included the corresponding baseline value of the MRI variable as a predictor. Additional details of the statistical analysis are in Results S1.

## Results

### Overview of Study Cohort

Tables 1, 2, 3 show the clinical, demographic and MRI features and environmental factor distributions of the cohort.

**Table 1:** Demographic, clinical and other characteristics of the cohort.

Characteristic	Value
Females: Males (% Female)	141: 70 (67%)
Age, years	28.9±7.8
Monosymptomatic onset	170/209 (81%)
Polysymptomatic onset	39/209 (19%)
Median EDSS (IQR) at baseline	1.50 (0.50)
Number of CEL at baseline	1.1±3.1
Number of T2-lesions at baseline	11.9±8.4
Volume of T2-lesions at baseline, cm^3^	5.1±5.9
*HLA DRB1* * *1501* positive	97/198 (49%)
Active smokers	66/194 (33%)
Anti-CMV positive	107/193 (55%)
Anti-EA-D positive*	18/193 (9.3%)
Anti-EBV EBNA-1:PositiveIn highest quartile	193/193 (100%)48/193 (25%)
Anti-EBV VCA:PositiveIn highest quartile	192/193 (99.5%)48/193 (25%)
Vitamin D status:Deficiency (<20 ng/ml)Insufficiency (≥20 to <30 ng/ml)Sufficiency (≥30 ng/ml)	143/185 (77%)34/185 (18%)8/185 (4.3%)

The continuous variables are expressed as mean ± SD and the categorical variables as frequency (%).

* Anti-EBV EA-D was not included in additional analysis because of the limited sample size of anti-EA-D positive group.

**Table 2:** Clinical and MRI characteristics at baseline and at 2-years.

Clinical or MRI Characteristic	Baseline	2-years	*p*-value*
Median EDSS (IQR)	1.50 (0.50)	1.50 (0.75)	0.17
Clinical progression	–	94/211 (45%)	
MRI progression	–	148/210 (71%)	
Clinical or MRI progression	–	169/211 (80%)	
Number of subjects with ≥ 1 relapses	–	90/211 (43%)	
Total number of relapses over 2-years	–	0.91±1.3	
Annual relapse rate	–	0.46±0.66	
Median time to first relapse^§^, months	–	5.7±8.0	
CE-lesion number	1.1±3.1	0.60±3.2	0.001
CE-LV cm^3^	0.098±0.34	0.063±0.43	0.004
T2-LV cm^3^	5.1±5.9	4.8±6.8	0.001
NBV cm^3^	1506±71	1479±74	<0.001
NWMV cm^3^	713±38	703±40	<0.001
NGMV cm^3^	793±47	776±49	<0.001
Cumulative CE-lesion number	–	1.0±4.1	
Number of new T2 lesions	–	3.50±7.9	
Number of new and enlarging lesions	–	4.80±11	
Change in brain volume %	–	−1.37±1.4	
Change in gray matter volume %	–	−1.69±2.2	

The continuous variables are expressed as mean ± SD and the categorical variables as frequency (%).

* Non-parametric Wilcoxon test. § For patients with one or more relapses.

**Table 3:** Baseline clinical, MRI and other characteristics and changes of patients progressing to CDMS in 2-years.

Clinical or MRI Characteristic	Not CDMS	CDMS	*p*-value*
Females: Males (% Female)	75:46 (62%)	66:24 (73%)	0.10
Age, years	30.1±7.7	27.2±7.6	0.002
Median EDSS (IQR)	1.50 (0.75)	1.50 (0.50)	0.53
Cumulative number of relapses	0	2.14±1.2	<0.001
Annualized relapse rate	0	1.07±0.59	<0.001
Baseline CE-lesion number	0.38±1.1	1.95±4.4	<0.001
Baseline CE-LV cm^3^	0.026±0.077	0.19±0.50	<0.001
Baseline T2-LV cm^3^	4.1±4.5	6.3±7.3	0.057
Baseline NBV cm^3^	1497±64	1516±79	0.034
Baseline NGMV cm^3^	786±44	802±49	0.007
Baseline NWMV cm^3^	712±36	714±42	0.62
Cumulative CE-lesion number	0.42±1.4	1.9±5.9	<0.001
Number of new T2 lesions	2.3±5.7	5.1±9.9	<0.001
Number of new and enlarging lesions	3.0±8.1	7.2±14	<0.001
Change in brain volume %	−1.08±1.2	−1.77±1.6	<0.001
Change in gray matter volume %	−1.35±2.0	−2.18±2.4	0.006
Change in white matter volume %	−0.45±2.8	−1.07±2.4	0.32
*HLA DRB1* * *1501* positive	53/115 (46%)	44/83 (53%)	0.39
Active smokers	39/111 (35%)	25/83 (30%)	0.54
Anti-CMV positive	52/111 (47%)	55/82 (67%)	0.006
Anti-EBV EBNA-1 in highest quartile	29/111 (26%)	19/82 (23%)	0.74
Anti-EBV VCA in highest quartile	27/111 (24%)	21/82 (26%)	0.87
Vitamin D deficiency	79/105 (75%)	64/80 (80%)	0.48

The continuous variables are expressed as mean ± SD and the categorical variables as frequency (%).

* Non-parametric Mann-Whitney test for clinical and MRI variables. Fisher exact test for genetic and environmental variables.

**Figure 1:**
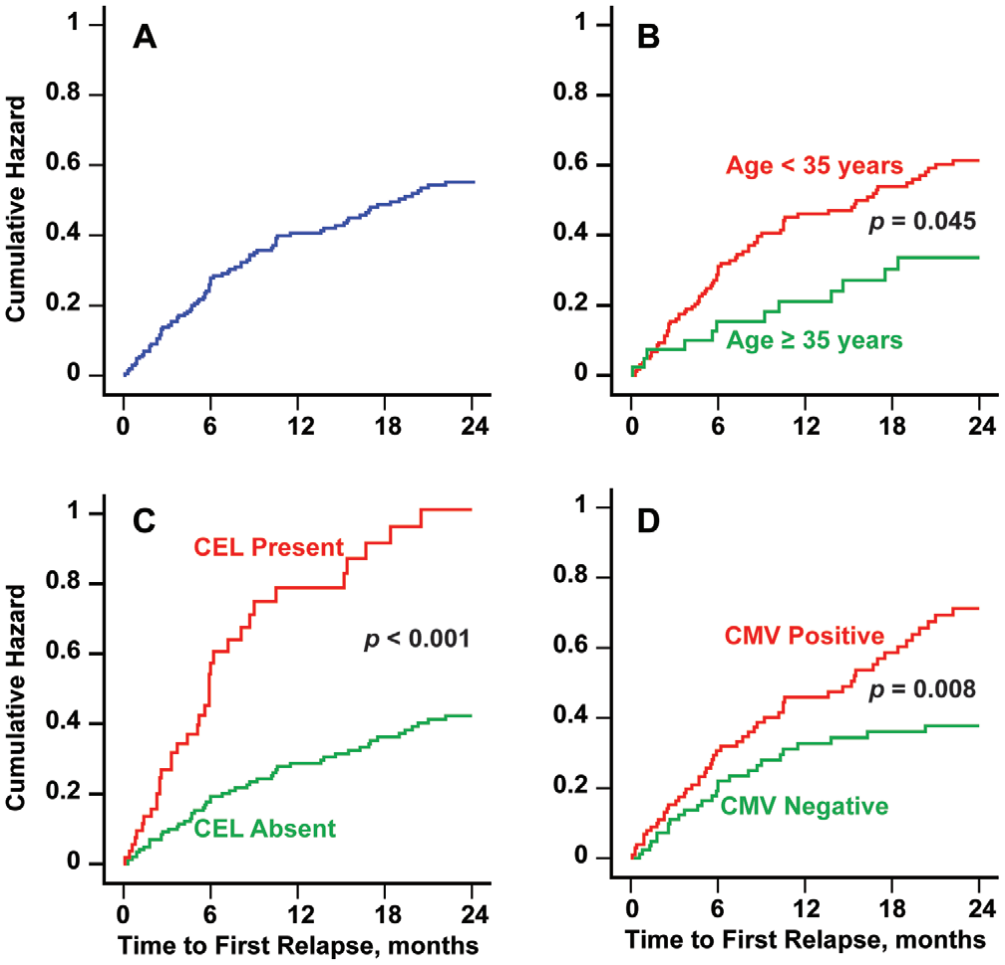
Dependence of time to first relapse on demographic, MRI and environmental factors. Figure 1A shows the cumulative hazard function for time to first relapse in all subjects. Figures 1B-D show the cumulative hazard functions for the age <35 years (red line) vs. age ≥35 years (green line), CEL present as baseline (red line) vs. CEL not present at baseline (green line) and CMV positive (red line) vs. CMV negative sub-groups, respectively. The corresponding covariate *p*-values from Cox regression are also shown.

During the 2-year period, 43% of patients (90 of 211) experienced relapses; the median time to relapse was 5.6 months. Figure 1A shows the cumulative hazard for time to the first relapse.

Based on EDSS change, 25 of 211 (12%) showed EDSS progression and 36 of 211 (17%) showed EDSS improvement (defined as ≥1 point decrease in EDSS at Month 24 compared to EDSS at baseline). Four patients (1.9%) showed EDSS progression but did not experience relapses during the 2-year period.

### Anti-CMV Positivity is Associated with Relapses

#### Associations of Anti-CMV Positivity with Relapses

Anti-CMV antibody positivity was associated as a trend with an increase in the number of relapses during the 2-year period (*p* = 0.014). The marginal mean for the number of relapses during the 2-year period was 0.52± SE 0.096 in the anti-CMV negative group compared to 0.90± SE 0.14 in the anti-CMV positive group.

Anti-CMV positivity was associated with progression to CDMS over the 2-year period (*p* = 0.004, Odds ratio  = 2.51, 95% confidence interval  = 1.35–4.68). In the anti-CMV antibody positive group, 51% (55 out of 107) experienced relapses compared to 31% (27 out of 86) in the anti-CMV antibody negative group.

Anti-CMV antibody positivity was associated with time to first relapse (Figure 1, *p* = 0.008, hazard ratio  = 1.9).

#### Associations of Other Factors with Relapses

Anti-EBV VCA highest quartile status, anti-EBV EBNA-1 highest quartile status, HLA DRB1*1501 positivity, active smoking status and vitamin D deficiency status were not associated with the number of relapses (all *p*>0.26), progression to CDMS (all *p*>0.44) or time to first relapse (all *p*-values >0.41).

However, younger age and female sex were associated with increased number of relapses over the 2-year period (*p*<0.001 for age and *p* = 0.021 for sex). Increased age (*p* = 0.009) and age ≥35 years status (*p* = 0.045, a trend, Figure 1) were associated with increased time to first relapse.

The presence (*p*<0.001) and number of CEL (*p* = 0.004) at baseline were also associated with the number of relapses. The presence of CEL at baseline was associated with decreased time to relapse (Figure 1, *p*<0.001). The number of T2 lesions at baseline was also associated with a trend toward decreased time to first relapse (*p* = 0.013).

### Anti-EBV VCA Antibodies are Associated with Disability Progression

Anti-EBV VCA in the highest quartile (*p* = 0.023, OR  = 2.84, 95% CI  = 1.16–6.96) was associated as a trend with EDSS progression. EDSS progression occurred for 11 of 49 (25%) patients in the highest quartile of anti-EBV VCA compared to 13 of 145 (9%) patients in the lower quartiles of anti-EBV VCA.

Anti-CMV positivity, anti-EBV EBNA-1 in the highest quartile, HLA DRB1*1501 positivity, active smoking status and vitamin D deficiency were not associated with EDSS progression (*p* = 0.063 for CMV positivity; all other *p*>0.46).

### Anti-EBV VCA Antibodies are Associated with MRI Progression

#### Contrast Enhancing Lesions

Anti-EBV-VCA highest quartile status was associated as a trend with increased CEL number (*p* = 0.031) but not CE-LV between baseline and 2 years (*p* = 0.31). The marginal mean ± SE for the highest anti-EBV VCA quartile was 0.49±0.15 lesions compared to 0.24±0.048 for the lower quartiles. The cumulative number of CEL over 2-years was associated as a trend with anti-EBV VCA highest quartile (*p* = 0.041, marginal mean ± SE  = 0.90±0.22 for the highest quartile vs. 0.51±0.077 for the lower quartiles).

Anti-EBV-EBNA-1 in the highest quartile was associated with increased CEL number (*p* = 0.003) but not CE-LV between baseline and 2-years (*p* = 0.21). The marginal mean ± SE for the highest anti-EBV EBNA-1 quartile was 0.52±0.14 lesions compared to 0.20±0.045 for the lower quartiles.

#### New and Newly Enlarging T2 Lesions

**Figure 2:**
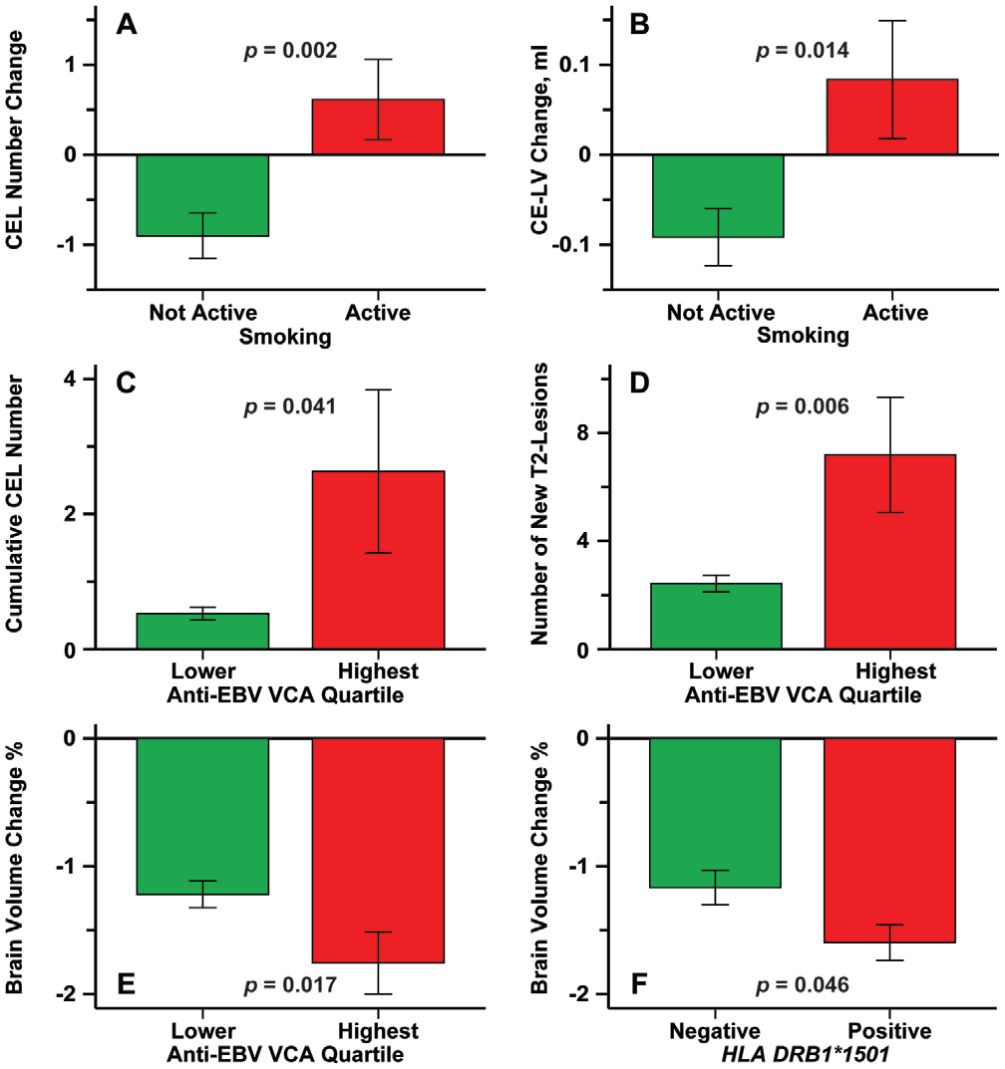
Dependence of change in individual MRI lesion-related variables over 2-years on the specific genetic or environmental factors. Figures 2A and 2B show the dependence of change in CEL number and change in CE-LV, respectively, on smoking status. Figures 2C shows the dependence of number of new and newly enlarging T2-lesions on anti-EBV VCA highest quartile status. Figure 2D shows the dependence of number of new T2-lesions on anti-EBV VCA highest quartile status. The bars represent mean values and the error bars are standard errors. The red colors denote the positive group and the green bars the negative group. The corresponding covariate *p*-values from regression are also shown.

Anti-EBV VCA highest quartile status was associated with a trend toward increased number of new and newly enlarging T2 lesions (Figure 2C, *p* = 0.018). The marginal mean for the highest anti-EBV VCA quartile was 4.53±0.83 lesions vs. 2.80±0.29 lesions for the lower quartiles.

The effect of anti-EBV VCA highest quartile status on the number of new and newly enlarging T2 lesions was explained by its effect on the number of new T2 lesions (*p* = 0.006). The marginal mean for the number of new lesions in the patients in the highest quartile of anti-EBV VCA levels was 3.56±0.67 lesions compared to 2.01±0.22 lesions for those in the lower quartiles.

#### Brain Atrophy

Anti-EBV VCA highest quartile status (*p* = 0.017, *r_p_* = −0.18) was associated with a trend toward decreased PBVC over 2-years.

### Smoking is Associated with Contrast Enhancing Lesions

Active smoker status was associated with increase in CEL number (*p* = 0.002, Figure 2A) and a trend toward increased in CE-LV between baseline and 2-year (*p* = 0.014, Figure 2B). The marginal mean number of CEL at 2 years in the active smoker group was 0.51±0.13 lesions compared 0.19±0.044 lesions in the non-active smoker group.

Active smoking status was not associated with the number of new and newly enlarging T2 lesions (*p* = 0.86) or brain atrophy as assessed by PBVC (*p* = 0.64).

### Vitamin D, Anti-CMV Positivity and HLA DRB1*1501 and MRI Progression

#### Contrast Enhancing Lesions

Anti-CMV positivity, HLA DRB1*1501 positivity and vitamin D deficiency were not associated with cumulative CEL number over 2-years, CEL number or volume at 2 years or with new or newly enlarging T2 lesions (all *p*-values >0.35).

#### New and Newly Enlarging T2 Lesions

A trend was found for increased number of new T2 lesions and vitamin D deficiency (*p* = 0.019, marginal means ± SE  = 2.54±0.25 new lesions in vitamin D deficient vs. 1.49±0.32 in the vitamin D insufficient or sufficient group).

#### Brain Atrophy

HLA DRB1*1501 positivity (*p* = 0.046, *r_p_* = −0.15) was associated with trend of decreased PBVC over the 2-year period. A weak trend was found for anti-CMV positivity (*p* = 0.054, *r_p_* = −0.14). We did not obtain evidence for significant associations of PBVC with anti-EBV EBNA-1 (*p* = 0.43) and vitamin D deficiency (*p* = 0.69).

### Effects of Risk Factor Combinations

These results are summarized in Results S1. Based on these analyses, we surmise that the anti-CMV positivity-anti-EBV VCA highest quartile combination is a parsimonious explanatory predictor because it exhibits stronger associations with more clinical and MRI variables.

## Discussion

In this study of CIS patients with oligoclonal bands in CSF and 2 or more lesions on the brain MRI as entry criteria, we examined the associations of environmental factors on disease progression. Relapses in CIS patients were associated with CMV positivity whereas anti-EBV VCA highest quartile status was associated with progression on MRI measures, including accumulation of CEL and T2 lesions and development of whole brain atrophy. We also investigated combinations of risk factors and found that the anti-CMV positivity-anti-EBV VCA highest quartile status combination was additively associated with both clinical and MRI outcomes.

Our findings of associations of anti-EBV VCA antibodies with brain atrophy are consistent with a previous study in an Italian patient group with MS [6]. In contrast, Lunemann *et* *al*. reported that only anti-EBV EBNA-1 antibodies were associated with conversion to CDMS [14] and found associations for new T2-lesions with both anti-EBV EBNA-1 and anti-EBV VCA antibodies. Our prospective study was conducted in cohort of high-risk patients on interferon-beta-1a treatment, whereas only 65.5% of the subjects in the Lunemann et al. study had oligoclonal bands [14]. Ingram *et* *al*. did not find associations between anti-EBV EBNA-1 IgG levels and disease activity in MS [25].

We did not obtain evidence that smoking was associated with decreased time to relapse as reported by Di Pauli *et* *al*. [15]. The discordance may be attributable to methodological differences: Di Pauli *et* *al*. [15] had 3-year follow-up and used a smoking questionnaire. We did not obtain patient smoking history but measured cotinine to objectively assesses active smoking status.

The genetics models for MS etiology implicate a network of immune related processes with HLA loci at the hub [26]. Although HLA DRB*1501 has been associated with low N-acetyl-aspartate (NAA) concentration in WM and WBV [27], numerous studies have reported lack of associations with MS disease severity [28]–[31]. Our finding of a trend between HLA DRB1*1501 positivity and decreased PBVC must be interpreted conservatively given its borderline *p*-value and the lack of associations with GM or WM atrophy and with CEL or T2-lesions, which are related to inflammatory activity.

Although vitamin D deficiency exhibited a trend with increased new T2 lesions, we did not find the associations with relapse rate that have been reported for MS [10]–[13]. We attribute the lack of evidence for vitamin D effects on relapse rate to the low frequency of vitamin D sufficiency in our study sample. Vitamin D supplementation, which is frequent among MS patients, is less likely in the younger CIS population.

The immediate early 1 (IE1) protein of CMV is an antagonist of Type 1 interferon but stimulates interferon-gamma responses via signal transducer and activator of transcription 1 (STAT1) [32]. This may provide a mechanistic framework for understanding our CMV findings in our interferon-beta treated cohort. We used the anti-CMV positivity anti-VCA highest quartile status combination risk factor score to assess the effects of the combination because a risk factor score is easily interpreted. EBV and CMV are both herpesviruses and evidence from transplant patients and immunological studies suggest that CMV infection can cause reactivation of EBV and alter cytokine production from immune cell populations [33]–[36].

We now discuss the strengths and weaknesses of our study. Although our study has a small sample size compared to genomewide association studies, it was conducted in the framework of a prospective, longitudinal, multi-center study and we had available clinical and MRI measures over a 2-year period. The relative homogeneity of the Czech patient sample and the single interferon-beta 1a treatment study design increases the power to detect effects. However, the homogeneity may potentially limit the ability to extrapolate the findings to other populations and to other treatments. Additionally, the results represent the contributions of environmental factors to the combined effects of both treatment and disease progression. The study inclusion criteria limited our ability to assess conversion according to the 2010 Polman-McDonald criteria [37].

In conclusion, our results suggest that environmental factors such as cigarette smoking, CMV exposure and responses to EBV may contribute to disease progression in interferon beta treated CIS patients. Smoking cessation and therapeutic and preventive interventions directed against CMV and EBV may potentially be useful in further slowing disease progression in this patient population.

## Supporting Information

Results S1 (PDF)
