# Supplementary material for: Environmental Factors Associated with Disease Progression after the First Demyelinating Event: Results from the Multi-Center SET Study
Source: PLoS One. 2013 Jan 8;8(1):e53996. doi: 10.1371/journal.pone.0053996 (PMC3540021; doi:10.1371/journal.pone.0053996)
Supplement: Results S1 — (PDF) [file pone.0053996.s001.pdf]

SUPPLEMENTARY RESULTS

MRI ACQUISITION AND ANALYSIS

Image Acquisition: MRI was performed on all patients using a 1.5 T magnet (Philips Gyroscan NT 15, Best, the Netherlands). Axial brain images were obtained using fluid attenuation inversion recovery (FLAIR) with 1.5-mm thickness (TR/TE/TI 11000/140 /2600 ms, matrix size 256 × 181, flip angle 90). Axial T1-weighted three-dimensional spoiled-gradient recalled (SPGR) images were obtained with 1-mm slice thickness (TR/TE 25/5 ms, matrix size 256 × 204, flip angle 30). Both FLAIR and SPGR images were non-gapped. In addition patients obtained post-contrast T1 spin echo (SE) 3-mm slice thickness scan 5 minute after contrast injection of a single dose of 0.1 mmol/kg of Gd-DTPA) with TE/TR = 12/450 ms.

Image Analysis: The scans were collected centrally at the Department of Radiology at Charles University (Prague, Czech Republic). All MRI scans were transferred and analyzed by the Buffalo Neuroimaging Analysis Center, Department of Neurology, State University of New York (Buffalo, New York, USA).

Lesion Measures: The T2- and contrast enhancing lesion (CEL) number and lesion volumes (LVs) were measured on FLAIR and on T1 post-contrast images, respectively, using a semi-automated edge detection contouring/thresholding technique previously described [1]. Using FMRIB's FLIRT [2], all follow-up FLAIR and T1-SE post-contrast images were co-registered to the baseline source images using a 6 degree-of-freedom rigid-body model. All subsequent lesion analysis was done using the co-registered images. For each time point, T2 lesion activity analysis was performed via the aid of a "subtraction image". Briefly, the FLAIR from the previous time point was subtracted from

the corresponding current image. The result was then smoothed with a Gaussian kernel of 0.5 mm. Cross-sectional regions of interest (ROIs) were overlaid on the subtraction image to facilitate the identification of new and enlarging T2-lesions.

Global and Tissue-Specific Atrophy Measures: For baseline analyses, the SIENAX cross-sectional software tool was used (version 2.6), with corrections for T1-hypointensity misclassification using an in-house developed in-painting program. Normalized whole brain volume (WBV), normalized gray matter volume (NGMV) and normalized white matter volume (NWMV) were measured as previously described [3].

For longitudinal changes of the WBV, we applied the SIENA method [4] to calculate the percentage brain volume change (PBVC). To quantify longitudinal GM and WM volume changes, we used a modified hybrid of FMRIB's SIENA and SIENAX tools. We used a brain- and skull-constrained co-registration technique to place both baseline and follow-up images into a joint space halfway between the two. Next, we combined baseline and follow-up intracranial volume masks via union, and valid voxel masks via intersection, ensuring that the same imaging volume was analyzed at both time points. Finally, we segmented the resulting images with a modified longitudinal version (L-FAST) of FMRIB's FAST tissue segmentation tool [5] that uses a 4-dimensional joint hidden random Markov field to prevent misclassification between time points when longitudinal intensity changes are lacking (or minimal). Total tissue volume was calculated for both baseline and follow-up for each tissue compartment from partial volume maps. The reproducibility of this analysis is similar to the SIENA method [4].

Data Analysis

SPSS (IBM Inc., Armonk, NY, version 19.0) statistical program was used for all

statistical analyses. In view of the multiple testing, a conservative *p*-value of ≤ 0.01 was used to assess significance; *p*-values ≤ 0.05 were considered to be trends.

Data Transformations: The homozygous and heterozygous *rs3135005* genotypes associated with *HLA DRB1*1501* positive allele were categorized as *HLA DRB1*1501* positive and the remaining homozygous *rs3135005* genotype was categorized as *HLA DRB1*1501* negative. Non-smokers were defined as those with cotinine ≤ 10 ng/ml and active smokers those with > 10 ng/ml. Subjects were considered anti-CMV positive if the relative concentration was greater than unity and anti-CMV negative if the relative concentration was unity or less.

The occurrence of anti-EBV EBNA-1 (100%) and VCA (99.5%) positivity was nearly ubiquitous in our study sample (Table 1). Because there are no established MS-relevant clinical standards for anti-EBNA-1 and anti-VCA levels, the anti-EBNA-1 and anti-VCA relative concentrations were categorized into quartiles, which are easier to interpret, using the observed quartile thresholds. Indicator variables for subjects in the highest quartiles of anti-EBNA-1 and anti-VCA levels were then obtained.

The raw 25(OH)VD_3 levels were logarithm transformed and deseasonalized using sinusoidal regression [6]. The form for the regression equation for 25(OH)VD_3 was:

$$\log 25(OH)VD_3 = a_0 + a_1 cos\left(\frac{2\pi(T-0.5)}{12}\right) + a_2 sin\left(\frac{2\pi(T-0.5)}{12}\right)$$

The *T* represents the month of blood draw (January = 1 to December = 12 scale with the 0.5 rounding values to the mid-point of the month). The a_0, a_1, a_2 are model parameters and were estimated from the data using non-linear regression. The deseasonalized values of log 25(OH)VD_3 were obtained by adding the residuals to the

model mean a_0. The deseasonalized values of 25(OH)VD_3 were calculated via the anti-log transformation. Vitamin D sufficiency (25(OH)VD_3 level ≥ 30 ng/ml) was found in only 4.3% of our study subjects. Therefore vitamin D levels were dichotomized based on the clinical threshold for vitamin D deficiency (25(OH)VD_3 level < 20 ng/ml) used by the Institute of Medicine [7]. The resultant distribution contained 77% of the sample in the vitamin D deficient group and 23% of the sample in the non-deficient group.

MRI progression was defined as the occurrence of one or more contrast-enhancing lesions or new T2 lesions during the 2-year period of the study. EDSS progression was defined as: i) an EDSS increase of ≥ 1.5 points at Month 24 for subjects with a baseline EDSS of 0, or ii) an EDSS increase of ≥ 1.0 at Month 24 for subjects with a baseline EDSS of ≥ 1.0. Clinical progression was defined as the occurrence of a relapse or EDSS progression. Sustained EDSS progression was not assessed because EDSS at Month 30 data are not available.

An indicator variable for the presence of CEL at baseline was also derived from the number of CEL at baseline.

To assess the impact of the combined effect of genetic and environmental risk factors, we created risk factor burden score defined as the sum of indicator variables for the constituent risk factors. Based on the results from univariate analysis, anti-CMV positivity and anti-EBV VCA in the highest quartile were selected as the initial risk factor pair of interest. The risk factor score was obtained by summing the indicator variables for anti-CMV antibodies and anti-EBV VCA antibodies. Subjects who were not positive for either risk factor were assigned a risk factor score of zero, those positive for only one risk factor were assigned a score of 1 and those positive for both anti-CMV antibodies

and for anti-EBV VCA antibodies were assigned a score of 2. In the next level of analysis, the *HLA DRB1*1501* and anti-EBV VCA risk factor pair was similarly assessed. Finally, *HLA DRB1*1501* was added to the anti-CMV positivity and anti-EBV VCA combination to create a risk factor score for the three predictors.

Analysis of Progression to CDMS, Relapses and Time to Relapse: The probability of progressing to CDMS was modeled with logistic regression. The time to first relapse was analyzed with the Cox proportional hazard model [8-10]. The number of relapses was analyzed with negative binomial regression. Age, sex and the genetic or environmental variable of interest were used as predictors in all the regression analyses.

Analysis of EDSS Progression: EDSS progression was analyzed as the dependent variable in logistic regression with age, sex and the genetic or environmental variable of interest as predictors.

Analysis of MRI Progression: The number of CEL at 2 years, the cumulative number of CEL over the 2-year period and cumulative number of new and newly enlarging T2-lesions over the 2-year period were each analyzed as dependent variables using negative binomial regression. The CE-LV at 2 years was assessed using Tweedie regression. The percent change in brain volume and percent change in GM and WM volumes over the 2-year period were analyzed as dependent variables with linear regression. All of the regression models contained main effects for age, sex, corresponding baseline value of the MRI variable and the genetic or environmental factor of interest as predictors.

COMBINATIONS OF ENVIRONMENTAL VARIABLES

RESULTS

Effects of Risk Factor Combinations

The initial risk factor combination was constituted with the anti-CMV positivity-anti-EBV VCA highest quartile pair because anti-CMV positivity was strongly associated with clinical measures, e.g., as number of relapses and time to relapse, whereas anti-EBV VCA in the highest quartile was associated with lesional and brain atrophy measures.

For the anti-CMV positivity-anti-EBV VCA highest quartile combination, 61 (32%), 109 (57%), and 23 (12%) of 193 patients had risk factor scores of 0, 1 and 2, respectively. The anti-CMV positivity-anti-EBV VCA highest quartile combination was associated with trends for increased risk of developing CDMS ($p = 0.012$), shorter time to relapse ($p = 0.011$), and higher number of relapses ($p = 0.018$) over the 2-year period. The combination was also associated with the risk of EDSS progression ($p = 0.002$): 5%, 12% and 36% of subjects with risk factor scores of 0, 1 and 2, respectively, had EDSS progression. The anti-CMV positivity-anti-EBV VCA highest quartile combination was associated with increased whole brain atrophy as assessed by the PBVC ($p < 0.001$, $r_p = -0.27$) and a trend toward increased number of new T2 lesions ($p = 0.018$). Figure S2 summarizes a subset of these results.

We also conducted additional regression analyses for PBVC that included both main effects and an interaction term for anti-CMV positivity and anti-EBV VCA highest quartile status. Because the interaction term did not reach significance ($p = 0.23$), the available data support a model wherein the contributions of anti-CMV positivity and anti-

EBV VCA highest quartile status to PBVC are additive.

We also examined the *HLA DRB1*1501* positivity-anti-EBV VCA highest quartile combination and the *HLA DRB1*1501* positivity-anti-CMV positivity-anti-EBV VCA highest quartile three-predictor combination. Associations with increased whole brain atrophy as assessed by PBVC were found for the *HLA DRB1*1501* positivity-anti-EBV VCA highest quartile combination ($p = 0.001$, $r_p = -0.25$) and also the *HLA DRB1*1501* positivity-anti-CMV positivity-anti-EBV VCA highest quartile combination ($p < 0.001$, $r_p = -0.30$). The *HLA DRB1*1501* positivity-anti-EBV VCA highest quartile combination was associated with increased number of new T2 lesions ($p = 0.010$). The *HLA DRB1*1501* positivity-anti-CMV positivity-anti-EBV VCA highest quartile combination was associated as a weak trend with the increased risk of developing CDMS ($p = 0.051$).

Based on these analyses, we surmise that the anti-CMV positivity-anti-EBV VCA highest quartile combination is a parsimonious explanatory predictor because it exhibits stronger associations with more clinical and MRI variables.

REFERENCES

1. Zivadinov R, Rudick RA, De Masi R, et al. Effects of IV methylprednisolone on brain atrophy in relapsing-remitting MS. Neurology 2001;57:1239-1247.

2. Jenkinson M, Bannister P, Brady M, Smith S. Improved optimization for the robust and accurate linear registration and motion correction of brain images. NeuroImage 2002;17:825-841.

3. Zivadinov R, Heininen-Brown M, Schirda CV, et al. Abnormal subcortical deep-gray matter susceptibility-weighted imaging filtered phase measurements in patients with multiple sclerosis: a case-control study. NeuroImage 2012;59:331-339.

4. Smith SM, Zhang Y, Jenkinson M, et al. Accurate, robust, and automated longitudinal and cross-sectional brain change analysis. NeuroImage 2002;17:479-489.

5. Zhang Y, Brady M, Smith S. Segmentation of brain MR images through a hidden Markov random field model and the expectation-maximization algorithm. IEEE transactions on medical imaging 2001;20:45-57.

6. Weinstock-Guttman B, Zivadinov R, Ramanathan M. Inter-dependence of vitamin D levels with serum lipid profiles in multiple sclerosis. Journal of the neurological sciences 2011;311:86-91.

7. Consensus Report. Dietary Reference Intakes for Calcium and Vitamin D. Food and Nutrition Board. Institute of Nutrition [online]. Available at: http://dietary-

supplements.info.nih.gov/factsheets/vitamind.asp.

8. Comi G, Martinelli V, Rodegher M, et al. Effect of glatiramer acetate on conversion to clinically definite multiple sclerosis in patients with clinically isolated syndrome (PreCISe study): a randomised, double-blind, placebo-controlled trial. Lancet 2009;374:1503-1511.

9. McCullagh P, Nelder JA. Models for Survival Data. In: Generalized Linear Models, 2nd ed. Boca Raton, Florida: Chapman & Hall/CRC, 2000.

10. Collett D. Modelling survival data in medical research. Boca Raton, FL: Chapman & Hall/CRC, 2003.

FIGURE LEGENDS

Figure S1. CONSORT diagram showing enrolled, included, and excluded patients.

Figure S2. Effects of risk factor combinations. Figure S2 shows the dependence of number of relapses over 2-years, the time to first relapse, number of new T2 lesions and percent change in brain volume over 2-years for the anti-CMV positivity-anti-EBV VCA highest quartile combination. The risk burden is shown on the *x*-axes of Figures S2 A, C, D and against the lines for Figure S2B: a value of 0 indicates that none of risk factors is positive (green), a value of 1 indicates that one of the two risk factors (orange) is positive and a value of 2 indicates that both risk factors are positive (red). The bars represent mean values and the error bars are standard errors. The covariate *p*-values from regression are also shown.

FIGURE S1

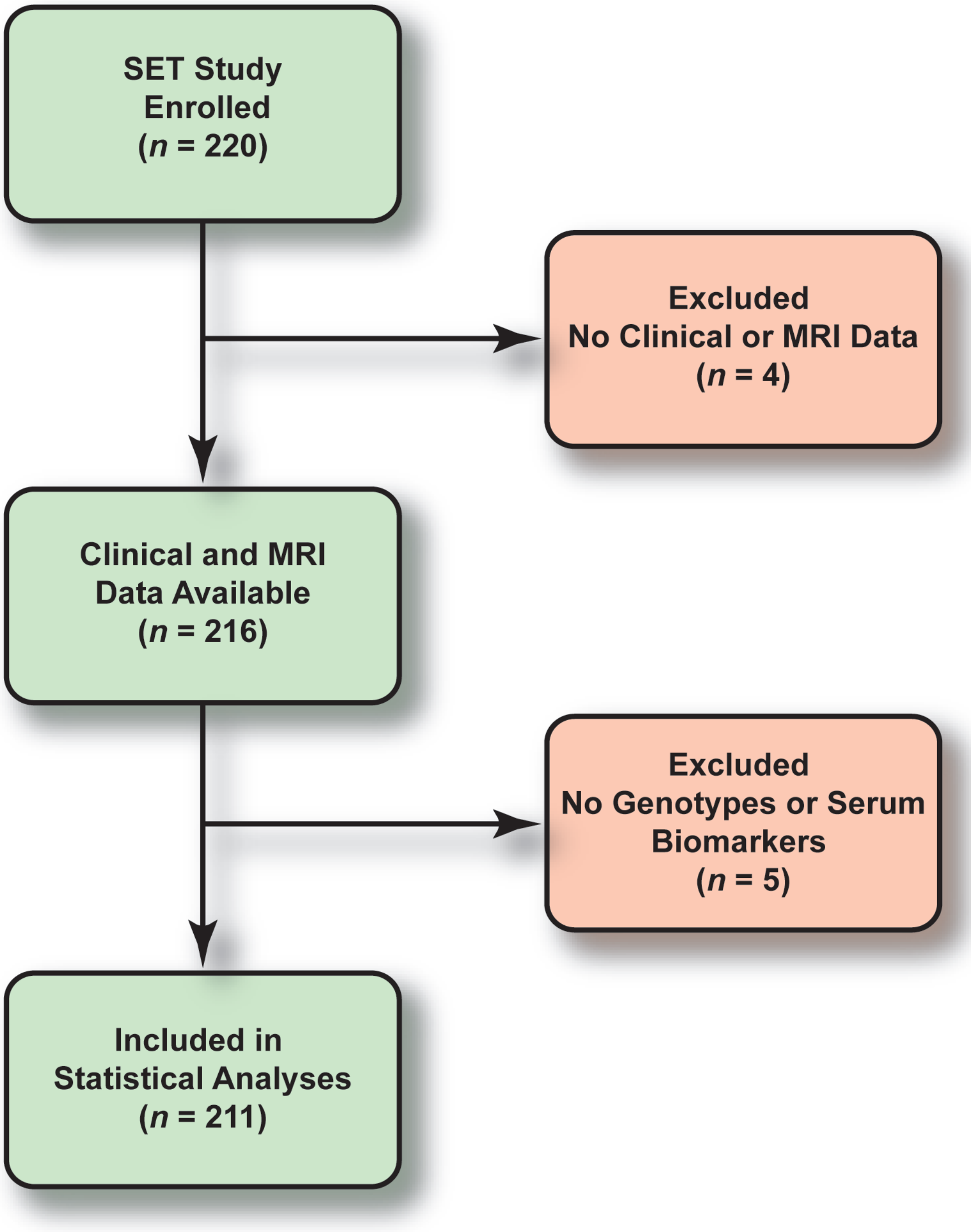
SET Study Enrolled (n = 220)
Excluded No Clinical or MRI Data (n = 4)
Clinical and MRI Data Available (n = 216)
Excluded No Genotypes or Serum Biomarkers (n = 5)
Included in Statistical Analyses (n = 211)

FIGURE S2

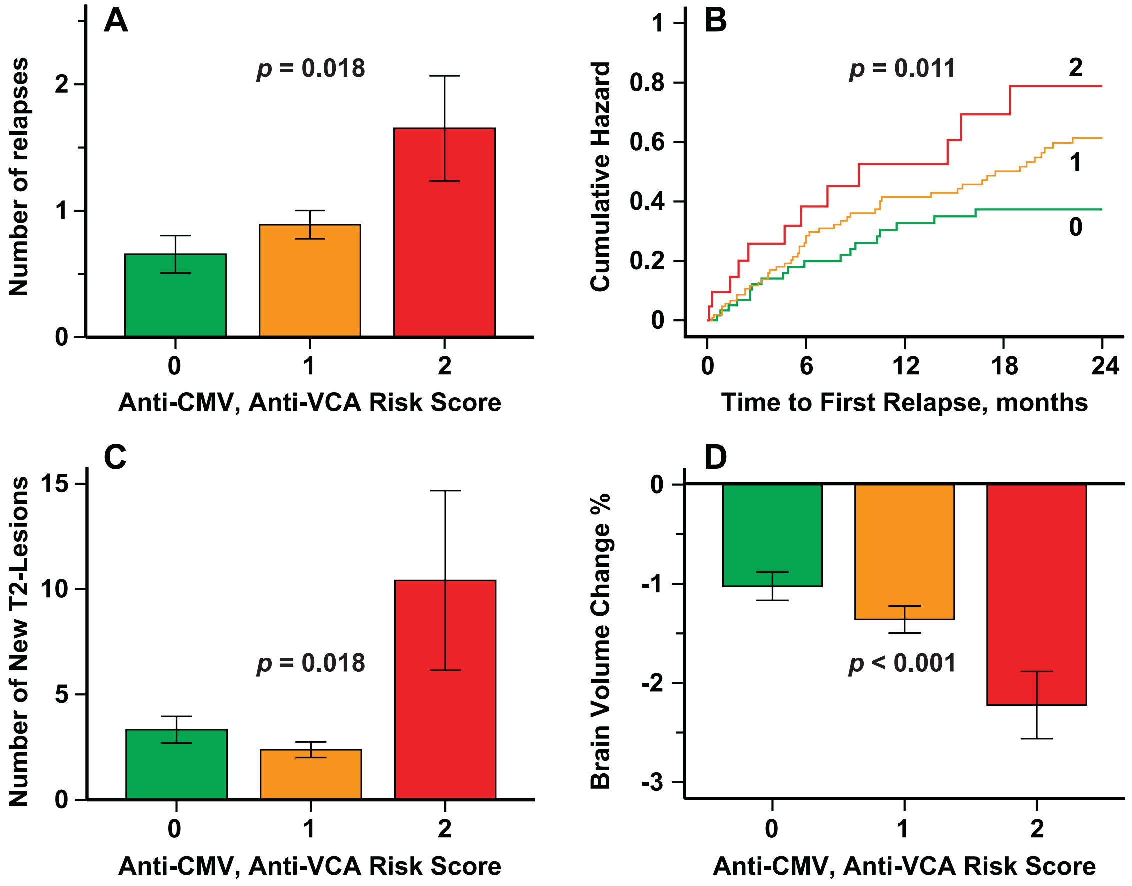

A
Number of relapses
p = 0.018
0
1
2
Anti-CMV, Anti-VCA Risk Score
B
Cumulative Hazard
p = 0.011
2
1
0
0.2
0.4
0.6
0.8
6
12
18
24
Time to First Relapse, months
C
Number of New T2-Lesions
p = 0.018
5
10
15
Anti-CMV, Anti-VCA Risk Score
D
Brain Volume Change %
p < 0.001
-1
-2
-3
Anti-CMV, Anti-VCA Risk Score
